# An appropriate treatment interval does not affect the prognosis of patients with breast Cancer

**DOI:** 10.1007/s44178-022-00010-z

**Published:** 2022-07-05

**Authors:** Wei Gao, Jiaxing Wang, Sifei Yin, Cuizhi Geng, Binghe Xu

**Affiliations:** 1 Department of Breast Cancer, Fourth Hospital of Hebei Medical University, 169 East Tianshan Avenue, Shijiazhuang, 050035 Hebei China; 2 Department of Chemical and Systems Biology, Stanford University, Stanford, California 94305 USA; 3 National Cancer Center/Cancer Hospital, Chinese Academy of Medical Sciences and Peking Union Medical College, Beijing, 100021 China

**Keywords:** Treatment delay, Risk evaluation, Prognosis, Breast cancer

## Abstract

**Purpose:**

Major public health emergencies may lead to delays or alterations in the treatment of patients with breast cancer at each stage of diagnosis and treatment. How much do these delays and treatment changes affect treatment outcomes in patients with breast cancer?

**Methods:**

This review summarized relevant research in the past three decades and identified the effect of delayed treatment on the prognosis of patients with breast cancer in terms of seeking medical treatment, neoadjuvant treatment, surgery, postoperative chemotherapy, radiotherapy, and targeted therapies.

**Results:**

Delay in seeking medical help for ≥12 weeks affected the prognosis. Surgical treatment within 4 weeks of diagnosis did not affect patient prognosis. Starting neoadjuvant chemotherapy within 8 weeks after diagnosis, receiving surgical treatment at 8 weeks or less after the completion of neoadjuvant chemotherapy, and receiving radiotherapy 8 weeks after surgery did not affect patient prognosis. Delayed chemotherapy did not increase the risk of relapse in patients with luminal A breast cancer. Every 4 weeks of delay in the start of postoperative chemotherapy in patients with luminal B, triple-negative, or HER2-positive breast cancer treated with trastuzumab will adversely affect the prognosis. Targeted treatment delays in patients with HER2-positive breast cancer should not exceed 60 days after surgery or 4 months after diagnosis. Radiotherapy within 8 weeks after surgery did not increase the risk of recurrence in patients with early breast cancer who were not undergoing adjuvant chemotherapy.

**Conclusion:**

Different treatments have different time sensitivities, and the careful evaluation and management of these delays will be helpful in minimizing the negative effects on patients.

## Introduction

The COVID-19 pandemic has affected the world since the beginning of 2020 and has strained the healthcare systems of many countries. Substantial medical resources have been redistributed in the efforts to fight the pandemic, thus causing disruptions in other hospital functions, including the treatment of patients with cancer [1]. Breast cancer is the most common malignancy in women [2] and affects 2.1 million patients worldwide [3]. Limited medical resources, government recommendations for staying indoors, and patients’ personal concerns about safety could all result in delays in seeking and providing care to patients with breast cancer. At the start of the COVID-19 pandemic, approximately 20% of patients with breast cancer experienced delays in at least one therapy modality [4]. As healthcare providers, we need to fully understand the effects of treatment delays on the prognosis of patients with breast cancer. This review aims to comprehensively summarize the effects of treatment delays and pauses on the outcomes of patients with breast cancer in multiple stages, including seeking medical treatment, neoadjuvant therapy, surgery, adjuvant chemotherapy, targeted therapy, and radiotherapy, and to provide treatment recommendations to doctors and patients.

## Delay in seeking medical treatment

In 1919, Farr [5] first proposed the concept of “delay in seeking help,” which is the period between an individual’s first awareness of a sign or symptom of illness and the initial medical consultation. In general, delayed help-seeking for 3 months or more is believed to have prognostic significance [6], and 3 months is generally used as a criterion for delayed help-seeking to calculate the prevalence [7]. Facione and Bish [7, 8] indicated that the rate of delayed help-seeking for patients with breast cancer in the United States is 15%–56%, with an average of approximately 20%–30%. After analyzing 25 years of data from a hospital in London, Richards [9] reported that the rate of delayed help-seeking in that region was approximately 32%. The rate of help-seeking reported in Germany [10], Malaysia [11], Iran [12, 13], and other countries was 18%–72.6%. In 1999, Richards [9] analyzed 2964 women who were diagnosed with breast cancer at Guy’s Hospital between 1975 and 1990. The duration of symptoms before hospital referral was recorded, and the median follow-up was 12.5 years. Ten years after the onset of symptoms, the survival rate was 52% for women with delays of < 12 weeks and 47% for those with longer delays. After 20 years, the survival rates were 34% and 24% for women with delays of < 12 weeks and for those with longer delays, respectively. The results showed that a longer delay in presentation was significantly associated with worse survival (*p* < 0.0001). Multivariate analyses indicated that the adverse effect of delays in presentation on survival was attributable to the association between longer delays and a more advanced stage. In the same year, Richards [14] reviewed and analyzed a total of 101,954 patients with breast cancer in 87 studies and reported that the 5-year survival rate of patients with delayed help-seeking of ≥3 months decreased by 12%. Longer delays are associated with more advanced American Joint Commission on Cancer (AJCC) stages, and there is a significant adverse relationship between longer delays and survival.

## Delay in surgical treatment

Surgery is an important part of breast cancer treatment. Most patients with breast cancer require surgical intervention, particularly patients with early stage breast cancer, in which neoadjuvant chemotherapy (NAC) is less commonly used, and surgery is the first intervention that patients receive.

Bleicher [15] analyzed the operation time of 94,544 patients in the Surveillance, Epidemiology, and End Results–Medicare-linked database and 115,790 patients with breast cancer in the National Cancer Database (NCDB). Both cohorts suggested that a period from diagnosis to surgery of > 30 days would reduce the overall survival (OS) of patients with breast cancer, and stratified analysis suggested more significance in patients with AJCC stages I and II. Polverini [16] analyzed 420,792 patients in the NCDB database with early breast cancer who underwent surgery and found that there was no difference in OS in patients with AJCC stage I cancer who underwent surgery within 8 weeks, patients with AJCC stage II cancer who underwent surgery within 12 weeks, and patients with AJCC stage I–II cancer who underwent surgery within 4 weeks. Patients with AJCC stage I cancer who were treated at 8 to 12 weeks (HR = 1.07; 95% CI 1.02–1.13) and > 12 weeks (HR = 1.19; 95% CI = 1.11–1.28) and patients with AJCC stage II cancer treated at > 12 weeks (HR = 1.16; 95% CI = 1.08–1.25) had decreased OS compared with patients treated at < 4 weeks. Why did both studies show that delayed surgical treatment has a greater effect on patients with early breast cancer? A possible explanation for this result is that for a patient, the time it takes for breast cancer to develop to stage II is longer than the time it takes to reach stage I. In these two studies, the patient’s breast cancer stage was determined at the time of diagnosis, and the effect of surgical delay on prognosis was analyzed. Therefore, for patients with stage I cancer, the same delay time accounted for a larger proportion of the total time from breast cancer to surgery than for patients with stage II cancer. When compared with patients in the same stage, the same delay time had a greater effect on patients with stage I cancer than on patients with stage II cancer. Eaglehouse [17] analyzed 9669 patients with breast cancer and found that a period from diagnosis to surgery of ≥36 days increased the risk for all-cause mortality. Multivariate analysis showed that women with a time to surgery of ≥36 days had a 30% higher risk of mortality than women with a time to surgery of 1–21 days. Swedish scholar Eriksson [18] analyzed the time interval of 7017 patients who underwent surgery after diagnosis and found that the risk of metastasis increased by 1.26 times in patients who underwent surgery more than 6 weeks after diagnosis.

Koca [19] compared patients with breast cancer who underwent hand therapy 1 year before the COVID-19 pandemic with those who underwent surgery 1 year after the start of the pandemic. Results showed that tumor size, axillary lymph node positivity, and NAC were all higher in patients who underwent surgery in the first year after the start of the pandemic than in those who underwent surgery before the pandemic. Khader [20] analyzed the time interval between diagnosis, surgery, and lymph node metastasis in 355,443 patients with cN0 breast cancer. After controlling for relevant factors, a one-month delay in surgery was associated with an increased likelihood of nodal positivity (OR = 1.04; 95% CI = 1.04–1.05) and decreased OS (HR = 1.03; 95% CI = 1.02–1.04). Compared with patients who underwent surgery less than 4 weeks after diagnosis, patients who underwent surgery more than 12 weeks after diagnosis had nodal positivity and relative risk of 5.3% (95% CI = 0.047–0.059) and 1.34 (95% CI = 1.30–1.38), respectively. This study suggests that delayed breast cancer surgery in cN0 patients is associated with an increased likelihood of axillary ascent and reduced survival rates.

The above studies suggest that delaying surgery for longer than 4 weeks might worsen patient outcomes.

## Delay in neoadjuvant therapy

Neoadjuvant therapy for breast cancer is mainly used for the preoperative treatment of patients with locally advanced cancer or breast cancer undergoing breast-conserving treatment. Only relevant data on NAC were found in this review.

Studies have been conducted on the effect of the time of NAC initiation on the prognosis of patients with breast cancer. Livingston-Rosanoff et al. [21] investigated whether delays in NAC initiation could affect patient survival. A total of 12,806 adult patients with breast cancer with AJCC stage I–III presentation in the NCDB were included in the analysis. The median time from the initial diagnosis to NAC initiation NAC was 4 weeks (range: 0–26). More than 90% of all patients started NAC within 8 weeks of diagnosis regardless of the receptor subtype. They found that there was no association between the time to initiation of NAC and patient survival for HER2-positive cancer (HR = 0.91; 95% CI = 0.74–1.12) and triple-negative cancer (HR = 1.10; 95% CI = 0.97–1.24). They concluded that in patients with triple-negative cancer or HER2-positive cancer, there was no association between NAC initiation within 8 weeks of diagnosis and patient survival.

Studies have analyzed the effect of time from the completion of neoadjuvant treatment (NAC in particular) to surgery on survival outcomes in patients with breast cancer. Arciero [22] conducted a retrospective analysis of patients undergoing NAC for AJCC stage I–III breast cancer between 1998 and 2010. The patients were divided into four subgroups according to the operation time after NAC: ≤4 weeks, > 4 and ≤ 6 weeks, > 6 and ≤ 8 weeks, and > 8 weeks. The median follow-up duration was 85 months. According to this study, the timing of surgical intervention following the administration of NAC may not affect the five-year disease-free survival (DFS) or OS between the subgroups. Sanford [23] analyzed 1101 patients with AJCC stage I–III breast cancer who received NAC before surgery. This study found no difference in the five-year local recurrence–free survival, relapse-free survival (RFS) (or recurrence-free survival), and OS among the three groups of patients with an interval of ≤4, 4–6, and > 6 weeks. Sensitivity analysis showed that OS did not worsen in patients who underwent surgery at ≤8 weeks. Other studies also suggested that surgery within 8 weeks after NAC does not affect prognosis [24].

Patients with locally advanced breast cancer require postoperative radiotherapy (PORT). How long is the appropriate interval between surgery and PORT? Silva [25] retrospectively analyzed 581 patients with breast cancer undergoing PORT at the Instituto do Cancer do Estado de Sao Paulo, with a median follow-up of 32 months (range: 2–82). A total of 43, 354, and 184 patients started PORT within 8 weeks, within 8–16 weeks, and more than 16 weeks after surgery, respectively. Research suggests that PORT initiation up to 8 weeks after surgery was associated with better DFS and OS in patients with locally advanced breast cancer who underwent NAC. However, the distribution of each group was uneven in this study. More than 90% of the patients received PORT after 8 weeks, and less than 10% of the patients received PORT within 8 weeks. In addition, the discussion section of the study also indicated that patients receiving PORT within 8 weeks had fewer events, such as cancer metastasis and death, which may have affected the results of the comparative analysis between groups.

## Delay in adjuvant chemotherapy

Studies have shown that adjuvant chemotherapy improves the survival of patients with breast cancer. However, does the timing of adjuvant chemotherapy for early breast cancer influence survival? Farolfi [26] conducted a multi-center phase III clinical study involving 921 patients with rapid proliferative early breast cancer. On the basis of ROC curve analysis, the study concluded that the best time for postoperative adjuvant chemotherapy is within 7 weeks. Shannon [27] analyzed the effect of the timing of adjuvant chemotherapy initiation in breast cancer. By using a prospectively maintained database, the study included a total of 1161 patients who underwent adjuvant chemotherapy for early breast cancer at Royal Marsden Hospital. The results showed that there was no significant difference in OS and DFS between patients receiving chemotherapy within 21 days and those receiving chemotherapy after 21 days. A prognostic analysis of 2782 patients by Sanchez [28] showed that there was no difference in in the 5-year OS of patients who started chemotherapy < 3 weeks, within 3–6 weeks, within 6–9 weeks, and > 9 weeks after surgery. In addition, some studies support the above conclusions [29, 30].

Some scholars believe that the delay in adjuvant chemotherapy will lead to varying degrees of survival in patients with breast cancer [31–34]. Gagliato [35] retrospectively analyzed 6827 patients with AJCC stage I–III breast cancer who received adjuvant chemotherapy at MD Anderson Cancer Center between 1997 and 2011. Among patients with stage II cancer, the study identified a detrimental effect on distant recurrence–free survival (DRFS) when chemotherapy was started ≥61 days after definitive surgery with no effect on OS. Among patients with stage III breast cancer, a delay of ≥61 days in the initiation of chemotherapy was associated with a detrimental effect on RFS, DRFS, and OS. Among patients with stage I cancer, there was no significant association between outcome and adjuvant chemotherapy. Downing [36] retrospectively analyzed the effect of the timing of adjuvant chemotherapy initiation on the survival of 10,366 patients with breast cancer from 1998 to 2004. The results showed that the 5-year OS was lower in patients with a delay of more than 10 weeks than in patients who received adjuvant chemotherapy within 3 weeks after surgery. In a study involving 24,843 patients with AJCC stage I–III breast cancer, Chavez-MacGregor [37] showed that patients who received adjuvant chemotherapy more than 12 weeks after surgery had a 27% increase in the risk of breast cancer death and a 34% increase in the risk of all-cause mortality. For every 4 weeks of delay in adjuvant chemotherapy, mortality increased by 4%.

Given the large amount of relevant research data, some scholars have conducted a large number of meta-analyses on this issue. Yu [38] performed a meta-analysis on the data of 34,097 patients with breast cancer from 1978 to 2013. The meta-analysis demonstrated that a four-week increase in the time to adjuvant chemotherapy was associated with a significant decrease in both OS and DFS. Similarly, Raphael [39] reported that for every 4 weeks of delay in adjuvant chemotherapy, the patients’ risk of death increased by nearly 5%. Biagi [40] conducted a meta-analysis on 15,327 patients with breast cancer. The meta-analysis demonstrated that a four-week increase in adjuvant chemotherapy was associated with an OS HR of 1.06 (95% CI = 1.02–1.10) and DFS HR of 1.08 (95% CI = 1.03–1.14). Therefore, starting adjuvant therapy 4 weeks after surgery is clinically significant.

Delayed chemotherapy has different effects on patients with breast cancer of different molecular types. Liu [41] found that delayed chemotherapy did not increase the risk of recurrence in patients with luminal A breast cancer, but delayed chemotherapy in patients with luminal B, triple-negative, or HER2-positive breast cancer without trastuzumab treatment reduced the five-year DFS. Morante [42] analyzed the relationship between the time of adjuvant chemotherapy initiation and prognosis in 687 patients with triple-negative breast cancer. The results showed that the OS and DRFS of patients with triple-negative breast cancer who started adjuvant chemotherapy at < 30 days after surgery were superior to those of patients who started adjuvant chemotherapy at ≥30 days after surgery. After investigating the reasons, it is believed that for patients with hormone receptor–positive tumors, the degree of benefit of adjuvant chemotherapy is not obvious [43]. By contrast, patients with high-grade and highly aggressive tumors will benefit from chemotherapy [43–45].

## Targeted therapy delay and prognosis in HER2-positive patients

Targeted therapy for patients with HER2-positive breast cancer is extremely effective and standardized and should be administered as soon as possible after diagnosis [46]. In a study on the effect of delayed adjuvant therapy on the prognosis of patients with breast cancer after surgery, Gagliato [35] indicated that patients with HER2-positive breast cancer who started adjuvant therapy with trastuzumab at ≥61 days after surgery had a significantly increased risk of mortality of more than 3 times compared with patients who started adjuvant therapy at ≤30 days after surgery. The five-year RFS and DRFS rates also decreased, but the difference was not statistically significant. In addition, patients with HER2-positive breast cancer who started adjuvant therapy with trastuzumab at 31–60 days after surgery showed no significant differences in OS, RFS, and DRFS compared with patients who started adjuvant therapy at ≤30 days after surgery.

Gallagher [47] investigated the effect of delaying the initiation of adjuvant trastuzumab therapy after breast cancer diagnosis on risk of relapse, OS, and RFS in a contemporary sample of patients with HER2-positive, nonmetastatic breast cancer. The study included 2749 patients with breast cancer who did not receive NAC: 79.9% initiated adjuvant trastuzumab within 6 months of diagnosis, and 20.1% initiated adjuvant trastuzumab at > 6 months after diagnosis. The results showed that patients who initiated trastuzumab at > 6 months after breast cancer diagnosis had a higher risk of five-year relapse, death, or relapse/death than those who initiated trastuzumab within ≤6 months of diagnosis. Specifically, when the targeted therapy delay was defined as > 4 months, patients who received trastuzumab for > 4 months (≤1 year) after breast cancer diagnosis still had a higher risk of five-year relapse/death than those who received trastuzumab for ≤4 months; however, the increase in the risk of relapse/death was lower than when the targeted therapy delay was at > 6 months after diagnosis.

The data in Gallagher [47] are specific and more reliable than the subgroup analysis in Gagliato [35]. Regarding the definition of delayed treatment, the calculation of delay time in Gallagher [47] starts with the diagnosis of breast cancer, whereas it starts with surgical treatment in Gagliato [35]. There is a time delay between diagnosis and surgical treatment. Therefore, the delay of targeted therapy does not exceed 60 days after surgery or 4 months after the diagnosis and has no significant effect on the prognosis of HER2-positive patients with breast cancer.

## Delay in PORT

Most studies that examined PORT delay in breast cancer have focused on the order of PORT and adjuvant chemotherapy and their interactions. A small number of studies investigated the effect of the interval from surgery to radiotherapy on the prognosis of patients without postoperative adjuvant chemotherapy. Nixon [48] retrospectively analyzed data from 653 patients with AJCC stage I or II pathologically node-negative breast cancer treated with breast-conserving surgery (BCS) without adjuvant systemic therapy. This retrospective analysis suggested that a delay of up to 8 weeks in the interval between BCS and the start of radiotherapy is not associated with an increased risk of recurrence in patients with early stage breast cancer treated with breast irradiation to at least 60 Gy without systemic therapy. Caponio [49] retrospectively analyzed data from 615 patients with breast cancer who were treated with BCS without adjuvant systemic therapy and divided them into 3 groups according to the timing of radiotherapy (≤60, 61–120, and > 120 days). The median follow-up time was 65.8 months. The results showed no significant differences in the distant metastasis–free survival (DMFS) and DFS between the three groups. Maaren [50] analyzed the prognosis of 2759 patients with primary invasive stage I–IIIA breast cancer who were treated with BCS and radiotherapy. Patients were divided into three subgroups according to the timing of radiotherapy after surgery, namely, < 42 days, 42–55 days, and > 55 days. This study showed that a time interval of > 55 days had better 10-year DFS and DMFS than a time interval of < 42 days; however, the 10-year OS was significantly lower for time intervals of 42–55 and > 55 days than for a time interval of < 42 days. The tumor size and tumor grade of patients in the < 42 days group were higher than those in the other two groups, and this finding may have an effect on DFS and DMFS. Therefore, the results suggest that radiotherapy within 6 weeks after surgery could provide a better OS. Ma [51] found that in patients with invasive breast cancer who did not require adjuvant chemotherapy after BCS, a longer delay in the initiation of PORT (≤69 vs. > 69 days) significantly decreased DFS (HR = 6.43). The 5-year cumulative incidence rates of disease recurrence were 3.0% for radiotherapy starting at ≤69 days after surgery and 12.6% for radiotherapy starting at > 69 days after surgery. This study also found that for patients with invasive breast cancer requiring adjuvant chemotherapy after BCS, the interval between the end of adjuvant chemotherapy and the start of adjuvant radiotherapy was associated with increased local recurrence rate, and patients with an interval of > 47 days had a higher 5-year local recurrence rate than those with an interval of ≤47 days (3.3% vs. 1.3%). This difference was more significant in the hormone receptor–negative subgroups.

## Summary

The outbreak and epidemic control of COVID-19 disrupts and delays the diagnosis and treatment of patients with breast cancer. This study comprehensively analyzed relevant studies and found the following: Delays in seeking medical help at ≥12 weeks affect the prognosis. Surgical treatment within 4 weeks of diagnosis did not affect patient prognosis. Starting NAC within 8 weeks after diagnosis, receiving surgical treatment at no more than 8 weeks after NAC completion, and receiving radiotherapy 8 weeks after surgery did not affect patient prognosis. Delayed chemotherapy did not increase the risk of relapse in patients with luminal A breast cancer. Patients with luminal B, triple-negative, and HER2-positive breast cancer who have not been treated with trastuzumab should receive adjuvant chemotherapy within 4 weeks. Targeted treatment delays in patients with HER2-positive breast cancer should not exceed 60 days after surgery or 4 months after diagnosis. Radiotherapy within 8 weeks of surgery did not increase the risk of recurrence in patients with early breast cancer without adjuvant chemotherapy. The COVID-19 epidemic has made the standardized diagnosis and treatment of breast cancer challenging. The rationalization of the diagnosis and treatment of patients with breast cancer while effectively preventing and controlling the COVID-19 epidemic, as well as achieving both antiepidemic and anticancer effects, is a key clinical issue during and after the COVID-19 epidemic.

## Data Availability

The datasets used and analyzed during the current study are available from the corresponding author on reasonable request.
